# Negative emotions and personal well-being among incarcerated filicide mothers in Rwanda

**DOI:** 10.1371/journal.pone.0271255

**Published:** 2022-07-27

**Authors:** Jean d’Amour Muziki, Thaoussi Uwera, Japhet Niyonsenga, Augustin Nshimiyimana, Siméon Gitimbwa Sebatukura, Jean Mutabaruka

**Affiliations:** 1 Department of Clinical Psychology, College of Medicine and Health Sciences of University of Rwanda, Kigali, Rwanda; 2 Department of Health Informatics, College of Medicine and Health Sciences of University of Rwanda, Kigali, Rwanda; 3 Mental Health & Behaviour Research Group, College of Medicine and Health Sciences of University of Rwanda, Kigali, Rwanda; Sapienza, University of Rome, ITALY

## Abstract

Despite the tremendous evidence of the harmful effects of maternal filicide on the lives of offenders, there is a scarcity on studies of their negative emotions and personal wellbeing especially in sub-Saharan Africa. Thus, this study was primarily aimed at assessing the prevalence of negative emotions experienced by filicide mothers and how they were associated with personal wellbeing in Rwanda. With an institutional-based cross-sectional study design, we measured the symptoms of anxiety, anger, shame, guilt, depression and personal well-being in a convenient sample of 55 filicidal mothers (mean age = 26.69; SD = 6.88) who were incarcerated in Nyarugenge prison. SPSS (version 24) was used to compute descriptive, Pearson correlation, independent t-test and regression analyses. The results indicated that the rates of shame were (100%), guilt (98.2%), anxiety (92.7%), depression (92.7%), low happiness and satisfaction with life (81.8%), and anger was (76.4%) in the current sample. Based on age category, there was no significant difference in anger scores, depression, guilt, shame and personal well-being scores between young and adult filicide mothers (p>.05). Young filicide mothers (*M* = 14.55, *SD* = 4.03), on the other hand, had higher anxiety scores than adult filicide mothers (*M* = 11.57, *SD* = 4.72), t = 2.52, p = .015. Finally, anxiety (β = -.507, t = -3.478, p = .001) and age (β = -.335, t = -2.685, p < .001) were negatively associated with personal well-being. The results emerged from this study highlight that filicide mothers experience substantial negative emotions and poor personal wellbeing regardless of their age category. However, poor personal wellbeing was associated with anxiety and age. Based on these results, mental health professionals should examine their mental state with respect to negative emotions and initiate programs that decrease the emotions as well as increase personal well-being.

## Introduction

Maternal filicide is the act of a mother killing her own child and is a major contributor to global child homicide rates [1]. Despite the new laws for child protection and penalties for filicide mothers, initiated and repeatedly updated in North America and England since the 17^th^ century [2] and later in 29 countries during the 20^th^ century [3, 4], the crime is still evident in every contemporary society [5] and is the leading cause of death among children [6]. Globally, approximately 205,153 children aged 0–14 years have been killed in the years between 2008 and 2017, and the majority of perpetrators were family members [7]. However, the number of intentionally killed adolescents and young adults (i.e. 15–29 years) was 1,691,869, which was the highest figure over the same period [7]. Though there is a general lack of scientific data on maternal filicide in several African countries, authors have revealed that more than 1,000 children die annually in South Africa and more than half of the deaths are due to child abandonment or other parental irresponsibility [8]. In Rwanda, data from the legal medicine of department of Kacyiru Hospital (a single hospital that carries out filicide related examinations in Rwanda) showed that the hospital had received 103 cases of filicide which was equivalent to nearly 67 cases per million in 2010 [9]. The most common method of filicide in Rwanda is suffocation, resulting from placing babies in boxes covered with linen and then either put in bushes or thrown in pits by mothers, only for passers-by to realize the corpses and then alert the police [9]. In Rwanda, legislation on infanticide is clear: Article 143 of the Rwanda Penal Code (2012) stipulates that “a person who kills his/her biological or adopted child shall commit infanticide and this shall be punishable by lifetime imprisonment”. However, there is no punishment at all for perpetrators who have psychiatric illness at the time of the offence [10].

A substantial body of literature from developed countries have highlighted that negative emotions like guilt and shame are experienced after committing a violent crime, but little is known in developing and post-conflict countries [11]. Similarly, another study has indicated that guilt and shame are long-lasting negative emotions that can be experienced within three years following the crime [12]. However, literature is still insufficient to inform us about other negative emotions such as anger [13], anxiety [14], and depression [15], and how they predict personal well-being [15] among filicidal mothers, especially in post conflict settings. Therefore, the present study is aimed at exploring the frequent negative emotions experienced by Rwandan mothers after committing the maternal filicide, and their associated personal well-being. In this study, negative emotion or negative affect is defined as an individual perception of a collective of adverse emotional states like worry, stress, shame, sadness, guilt, envy, depression, anxiety, anger, etc. [15]. Personal well-being is defined as an individual part of quality of life, a satisfactory, good and optimal state of personal life or nature [16]. It is also a complex concept that includes emotional well-being, positive functioning, resilience, self-esteem, a happy life and vitality [16].

Previous findings revealed a decline in general functioning, a lower level of personal well-being and a lower level of satisfaction with life in the sample of people suffering from various degrees and forms of anxiety [17]. It was noted that ineffective ways of dealing with anger, such as constant anger suppression, extra punitive anger outbreaks, frequent aggressive-expressive and increased excitability, have adverse effects on personal wellbeing [13]. Consistently, a recent study has indicated that negative emotions could be considered as an independent factor or predictor of personal wellbeing [18].

## Rationale of the study

While the vast majority of studies suggest that women are emotionally distressed before they kill their children [19–23], little is known about whether this emotional distress persists after one year post-offence and how it is associated with personal wellbeing. Parental pre-existing psychiatric disorders have been repeatedly found as the leading motives for filicides but are also evident in post offence period [20, 24]. In a qualitative study, Stanton and Simpson [24] found that the women avoided thoughts about the offense and their memories were often described as patchy and horrifying in nature after a long-period post-offence. According to Stanton and Simpson [24], the women described intense self-loathing and self-criticism which may have emerged from negative emotions. This study aimed to explore the long-lasting negative emotions^_^ i.e., anger, anxiety, depression, guilt, and shame and their associations with personal wellbeing among Rwandan incarcerated filicide mothers. By conducting this study, we contribute to the scientific community and organizations working with incarcerated filicide mothers in Rwanda, “a post-conflict country” and similar settings by initiating effective interventions strategies.

## Methods

### Study design

This study used institutional-based cross-sectional study design. It was conducted in Nyarugenge Prison which is in Nyarugenge District, the city of Kigali. Data collection was conducted from February 1 to March 20, 2020. We reached the participants during working hours from Monday to Friday (between 8 a.m. and 5 pm.).

### Sample and procedures

A convenient sample of 55 filicidal mothers aged at least 18 (mean age = 26.69; SD = 6.88) was selected from NYARUGENGE prison to participate in this study. Participants were recruited in collaboration with a social worker of the prison. Inclusion criteria included having committed maternal filicide one year ago or more, being incarcerated, and willing to participate. Individuals were excluded if they had severe mental disorder or chronic illness that would affect their judgement (this group was considered psychologically unstable because they were on psychotropic medication at the time of data collection).

Ethical approval was obtained from the Institutional Review Board of the University of Rwanda, CMHS (IRB-CMHS). After a thorough explanation of the study, the participant provided consent forms. In accordance with distress protocol, two research assistants were there to provide emotional support to the participants with emotional challenges, and later refer them to prison social workers for follow-up. The participants were assured that it was their right to withdraw from the study if they didn’t want to participate or if they changed their mind. Measures of anxiety, depression, shame, guilt, anger and personal well-being were translated into Kinyarwanda and back translated, respectively, by three and two psychologists who have proficiency in Kinyarwanda and English.

### Measurements

**Generalized Anxiety Disorder 7 (GAD-7)** consists of 7 items measuring worry and anxiety symptoms; the tool was initially developed starting from 13 items based on the criteria for GAD in the DSM-IV and other items in anxiety measures [25]. The respondents rated each item on a four-point Likert scale ranging from 0 (not at all), 1 (several days), 2 (more than half the day) to 3 (nearly every day). The total scores range from 0 to 21 with higher scores reflecting higher anxiety severity. GAD symptoms are considered clinically significant if the scores exceeds 10 [25]. The Cronbach’s alpha was 0.77 in the current sample.

**Novaco Anger Scale (NAS)** was a 25-item self-report questionnaire composed of cognitive, arousal, and behavioural subscales measuring a person’s disposition for anger [26]. The respondents were asked to rate the degree to which the incident described on the questionnaire would make them angry or provoke them on a Likert scale ranging from 0 (Very Little), 1 (Little), 2 (Moderate Amount), 3 (Much) and 4 (Very Much). The total score ranging from 0 to 45 stands for no significant symptoms of anger, 46 to 55 for being more peaceful than others, 56 to 75 for reacting with anger to life annoyances, 76 to 85 for being more irritable than others and 86–100 for frequent, long-term and intense furious reactions. The Cronbach’s alpha was 0.90 in the current sample.

**Severity Measure for Depression-Adult** (adapted from the Patient Health Questionnaire–9 [PHQ-9]) is a 9-item self-report questionnaire used to assess the severity of depressive symptoms in people over the age of 18 [27]. Each item was rated on a 4-point scale: 0 = Not at all, 1 = several days, 2 = more than half the days, and 3 = nearly every day. For total score interpretation, 0–4 = no significant symptoms of depression, 5–9 = significant symptoms of mild depression, 10–14 = significant symptoms of moderate depression, 15–19 = significant symptoms of moderately severe depression and 20–27 = significant symptoms of severe depression. The Cronbach’s alpha was 0.86 in the current sample.

**The State Shame and Guilt Scale (SSGS)** [28], is a 15-item self-rated measure of state feelings of shame, guilt and pride (5 items for each subscale). For this study, 5 items from the state’s feeling of pride subscale were removed from the tool. Each item was rated on a 5-point Likert scale ranging from 1 (“Not feeling this way at all”) to 5 (“Feeling this way very strongly’). The Cronbach’ alpha was 0.75 for state feelings of shame and was 0.83 for the guilt subscale. A total score range of 6–25 indicated feelings of shame or guilt, while a 1–5 score indicated the opposite [28].

**Personal Well-Being Index for Adult (PWI)** [18] **is a 7-items questionnaire** that measures satisfaction with the following life domains: “standard of living, health, life achievement, personal relationships, personal safety, community connectedness, and future security”. Each item was scored from 0 to 10 points (0 = no satisfaction at all; 10 = very satisfied). Items can be scored individually to derive a score for the corresponding domain, or all the scores for all items can be summed up and averaged to form the Personal Wellbeing Index (PWI). A total score range of 0–49 = indicates that there is no normative range and a score ranges of 50–100 = indicates that there is a normative range. The Cronbach’s alpha was 0.84 in the current sample.

### Statistical analysis

The Statistical Package for Social Sciences (SPSS version 24) was used to compute descriptive statistics, comparative, and multiple regression analyses, with a p-value of α = 0.05 retained. The independent t-test was used to examine whether there was significant difference in symptoms of anxiety, depression, guilt and shame between young mothers and adult mothers. Furthermore, tools’ reliabilities (internal consistencies) were tested by using Cronbach’s alpha, and Pearson’s correlation was used to examine inter-correlations between variables. Before computing multiple regression analysis (MRA), the following four main assumptions of this analysis were fully taken into account. We verified normal distribution, homoscedasticity, and multicollinearity (the tolerance value ≥ 0.2), after removing significant outliers. In this study, the predictor variable had tolerance values ≥ 0.37, thus excluded multicollinearity. Despite the compelling evidences showing that the main assumptions of MRA were not violated, the authors were still worried about the sample size (n = 55) and the way it could affect the statistical power [29]. However, the sample size of this study was greater than 30 which has recently been proven as the minimum sample for performing multiple regression analysis [30, 31].

## Results

### Socio-demographic data

The mean age of the study participants was 26.69 years (SD = 6.8) and the sample was predominantly dominated by individuals aged 21 to 25 years (49.1%). Before incarceration, most of the participants (30.9%) resided in Kigali city (**Table 1**). Moreover, most of the participants were single (80%) at the time of committing filicide, 5.5% were legally married, and the least were widowed (2%). For education level, only 36.3% of the study participants had attended secondary school while 10.9% of them had not attended school (illiterate). The most predominant professions in this sample were cultivation (16.4%) and students (16.4%).

**Table 1:** Characteristics of participants.

Variables		Frequency	Percent
**Age**			
	18–20	7	12.7
	21–24	20	49.1
	25–30	15	14.5
	31–35	7	12.7
	36–40	4	7.3
	41 and above	2	3.6
	**Total**	**55**	**100**
**Area of origin**			
	The city of Kigali	17	30.9
	Eastern province	12	21.8
	Western province	10	18.2
	Northern province	5	9.1
	Southern province	11	20
	**Total**	**55**	**100**
**Marital status**			
	Single	44	80
	Legally married	3	5.5
	Illegally married	4	7.3
	Widowed	1	1.8
	Separated	3	5.5
	**Total**	**55**	**100**
**Level of education**			
	Lower primary school	8	14.5
	Upper primary school	21	38.2
	TVET	3	5.5
	Lower secondary school	4	7.3
	Upper secondary school	13	23.6
	I didn’t attend school	6	10.9
	**Total**	**55**	**100**
**Previous occupation**			
	Unemployed	8	14.5
	Cultivator	9	16.4
	Housemaid	13	23.6
	Vendor	7	12.7
	Waitress	3	5.5
	Tailor	2	3.6
	Student	10	18.2
	Casual worker	3	5.5
	**Total**	**55**	**100**

### Rate of negative emotions and feelings among filicide mothers

As presented in Table 2, the participants had clinically significant levels of negative emotions and feelings like anger (76.4%), guilt (98.2%), shame (100%), depression (92.7%), and GAD (92.7%). The participants also had a lower personal wellbeing index (81.8%).

**Table 2:** Rates of negative feelings and satisfaction about life among participants.

Level of feelings of anger	Frequency	Percent
Anger and frustration is remarkably low (0–45 scores)	13	23.6
Considerably more peaceful than other participants (46–55 scores)	10	18.2
Reacting with anger to life’s annoyances (56–75 scores)	19	34.5
More irritable than other participants (76–85 scores)	8	14.5
Frequent, long-term and intense furious reactions (86–100 scores)	5	9.1
Total	55	100
**Feelings of guilt**	**Frequency**	**Percent**
No state feeling of guilt (1–5 scores)	1	1.8
State feeling of guilt (6–25 scores)	54	98.2
Total	55	100
**Feeling of shame**	**Frequency**	**Percent**
State feeling of shame (6–25 scores)	55	100
**Clinical Significant levels of depression**	**Frequency**	**Percent**
no depressive symptoms (0–4 scores)	4	7.3
Mild depression (5–9 scores)	12	21.8
Moderate depression (10–14 scores)	10	18.2
Moderately severe depression (15–19 scores)	12	21.8
Severe depression (20–27 scores)	17	30.9
Total	55	100
**Clinical Significant levels of GAD**	**Frequency**	**Percent**
No **GAD** (1–4 scores)	4	7.3
Mild **GAD** (5–9 scores)	8	14.5
Moderate **GAD** (10–14 scores)	23	41.8
Severe **GAD** (15–21 scores)	20	36.4
Total	55	100
**Personal wellbeing index**	**Frequency**	**Percent**
No normative range (0–49 scores)	45	81.8
Normative range (50–100 scores)	10	18.2
Total	55	100

### Comparison of negative emotions in young and adult filicide mothers

As shown in Table 3, an independent t-test demonstrated that there were no statistically significant differences in symptoms of anger (t (53) = -.528, p = .600), guilt (t (53) = .694, p = .490), shame (t (53) = -.219, p = .828), depression (t (53) = -.878, p = .384), and personal wellbeing between young and adult filicide mothers. However, there were statistically significant differences in GAD symptoms between young and adult filicide mothers (t (53) = 2.52, p = .015). According to the WHO, young age is a period ranging from 15–24 years, and adulthood ranges from 25–65 years.

**Table 3:** Independent samples test differential analysis in levels of negative emotions between young and adult filicidal mothers.

					Levene’s Test for Equality of Variances		t-test for Equality of Means		
Negative emotions	Groups of participants	N	Mean	SD	F	Sig.	t	df	Sig. (2-tailed)
**NAS**	Young filicidal mothers	27	57.59	18.19	317	.576	-.528	53	.600
	Adult filicidal mothers	28	60.35	20.54					
**SSGS-G**	Young filicidal mothers	27	22.33	4.03	1.164	.286	.694	53	.490
	Adult filicidal mothers	28	21.46	5.15					
**SSGS-S**	Young filicidal mothers	27	19.14	5.26	.320	.574	-.219	53	.828
	Adult filicidal mothers	28	19.46	5.44					
**SMD**	Young filicidal mothers	27	15.74	6.38	.549	.462	.878	53	.384
	Adult filicidal mothers	28	14.17	6.79					
**GAD-7**	Young filicidal mothers	27	14.55	4.03	1.33	.25	2.52	52.23	.015
	Adult filicidal mothers	28	11.57	4.72					
**PWI**	Young filicidal mothers	27	35.92	16.90	.132	.718	1.378	53	.174
	Adult filicidal mothers	28	29.42	18					

### Inter-correlations between variables (N = 55)

The results showed that there was a strong correlation between variables in general (Table 4). In the current sample, guilt was strongly correlated with shame (r = .78, p < .001), anger (r = .37, p = .005) and depression symptoms (r = 0.296, p = 0.28). Anger was correlated with shame (r = .374, p = .005), guilt (r = .290, p = .032), and GAD (r = .294, p = .02). Furthermore, depression symptoms were correlated with anxiety (r = .350, p = .009). Despite a significant correlation between the independent variables, the personal wellbeing index as a dependent variable was only related to GAD symptoms (r = -.459, p < .001).

**Table 4:** Correlation among the measured negative emotions and feelings.

	NAS	SSGS-G	SSGS-S	SMD	GAD-7	PWI
**NAS**	1	.290*	.374**	0.162	.294*	-0.199
**SSGS-G**		1	.783**	.296*	0.239	-0.041
**SSGS-S**			1	0.2	0.012	0.054
**SMD**				1	.350**	-0.247
**GAD-7**					1	-.459**
**PWI**						1

*.p<.05

**p<.01 (2-tailed).

**Note that**: NAS: Novaco Anger Scale; SSGS-G: the State Shame and Guilt Scale- Guilt subscale; SSGS-S: the State Shame and Guilt Scale- Guilt subscale Generalized Anxiety Disorder 7; PWI: Personal Well-Being Index;.

### Negative emotions and feelings as predictors of personal well-being

When age, anger, guilt, shame, depression and GAD were entered, the overall goodness of fit of the fitted regression model was significant F (6, 48) = 4.056, p = .002). The results showed that generalized anxiety disorder (β = -.507, t = -3.478, p = .001) and age (β = -.335, t = -2.685, p < .001) predicted personal well-being index. Anger (β = -.024, t = -.174, p = .863), guilt (β = .065, t = .314, p = .755), shame (β = .075, t = .356, p = .723), and depression symptoms (β = -.084, t = -.644, p = .523), however, did not predict personal wellbeing index in the current sample (Table 5).

**Table 5:** Effect of negative emotions and feelings on personal well-being.

	Unstandardized Coefficients		Standardized Coefficients	t	Sig.
Model	B	Std. Error	Beta		
**(Constant)**	75.23	13.78		5.45	.000
**Age**	-.859	.320	-.335	-2.685	.010
**NAS**	-.022	.126	-.024	-.174	.863
**SSGS-G**	.247	.788	.065	.314	.755
**SSGS-S**	.248	.697	.075	.356	.723
**SMD**	-.224	.348	-.084	-.644	.523
**GAD-7**	-1.937	.557	-.507	-3.478	.001

a. Dependent Variable: Personal well-being

## Discussion

This study was mainly aimed at assessing the prevalence of negative emotions and their associations with the personal wellbeing index among filicide mothers incarcerated in Nyarugenge prison. Our findings showed that the participants reported clinically significant levels of anger (76.4%), guilt (98.2%), and shame (100%). Consistently, authors have indicated that guilt and shame are key indicators of post loss psychopathologies [32]. Previous study have also shown that young mothers who committed infanticide can experience symptoms of anger [18]. Interestingly, this study revealed that there were no significant differences in symptoms of anger, guilt, and shame between young filicide mothers and adult filicide mothers. These results suggest that maternal filicide affects the emotions and feelings of perpetrators regardless of their age category.

Furthermore, significant symptoms of guilt and shame among women who lost their loved ones, were found to be predictors of severe symptoms of grief following that loss [33]. The feelings of self-blame felt can be explained by a variety of factors including lack of positive emotions in the absence of a deceased one, self-accusation, or being unable to prevent the death [34]. Recently, it was demonstrated that guilt of bereavement guilt is dependent on complicated grief, traumatic reactions, and poor physical health [35]. The high rate of shame is caused by a variety of psychopathologies including suicidal thoughts, high intensity of symptoms of anxiety and depression symptoms [36].

This study indicated high clinically significant levels of depression and generalized anxiety disorder in the current sample. Similarly, different studies have highlighted the elevated prevalence of depression [37, 38] and anxiety disorders [39] among filicide mothers. However, the rate reported in this study is quite higher than those reported in prior studies. This difference can be explained by the long-lasting effects of the Genocide on Rwandan mental and physical health. It was found that PTSD and its associated major depression and anxiety, somatic symptom disorders, hearing or speech loss, fainting and hiccups are still public health issues in Rwandans due to the genocide against the Tutsi in 1994 [40]. This study revealed that young filicide mothers suffered from higher anxiety symptoms than adults.

Furthermore, the participants of the current study reported a remarkable low personal wellbeing index. This rate may be an indicator of negative emotions like shame, guilt, depression, anger, anxiety and little joy [41]. Several scholars have revealed that in some societies, serious social punishments are imposed on women who give birth and neglect their children. They are frequently humiliated and rejected by their respective communities. These mothers are at high risk of being stigmatised in addition to suffering from anxiety, guilt and shame due to the new baby [42]. They are living in extreme poverty which limits their ability to support their children, get appropriate housing, and other basic human needs that may probably affect their personal wellbeing [43]. In this study, there was no significant difference in personal well-being index scores between young and adult filicidal mothers.

Personal wellbeing in this study was predicted by anxiety symptoms and age. However, personal wellbeing was not associated with anger, shame, guilt or depression symptoms in the current sample. Despite the lack of literature on associations between negative emotions and personal wellbeing in filicide mothers, our findings are in congruence with the ones of the study conducted on a sample of child care providers [44]. Personal wellbeing was negatively linked to anxiety related stress characterised by sleep disturbances, physical health problems and exhaustion [44]. Prior studies also showed that personal wellbeing was lower among the individuals aged 45 to 64 years [45, 46].

## Study limitations

This study had several limitations. First, this study was limited by a lack of investigating the role of the incarceration period in worsening negative emotions and personal wellbeing. Therefore, future studies should compare these negative feelings and personal wellbeing between incarcerated mothers who had committed such violent crimes and those who have not, to strengthen the conclusion and inferences made from the findings of the current study. Second, negative emotions such as grief, sadness, and loneliness that might be prevalent in the current sample were not explored. Therefore, future studies especially qualitative ones are warranted. Third, as this study was cross-sectional in design, we couldn’t distinguish whether the emotional distress in the current sample was from the period before the killing or after the killing. However, being incarcerated implies that the participants had no psychiatric illness at the time of the offence. This may indicate a likelihood of developing emotional distress in the post-offence period but longitudinal studies with large sample sizes are recommended for further insights. Fourth, secondary gain and malingering in incarcerated population were not considered in this study. Finally, all the tools were self-reported that would lead to response biases due to social desirability and they were not validated for the study population. However, the Cronbach’s alpha did show that the internal consistencies of the tools used in this study were in the acceptable range.

## Conclusion

Despite the study limitations, our findings indicate a high rate of negative emotions and a low rate of personal wellbeing in the sample of filicidal mothers. Personal well-being was negatively associated with anxiety symptoms and age. These results suggest that treating or significantly decreasing symptoms of anxiety may lead to considerable increase of personal well-being in filicide mothers with particular attention to adult mothers. Therefore, more support for the incarcerated filicide mother’s psychosocial well-being can help them have a better personal wellbeing index.

## Supporting information

S1 Dataset (SAV)

S1 Table Reliability and validity of questionnaires. (DOCX)
