# Supplementary material for: Negative emotions and personal well-being among incarcerated filicide mothers in Rwanda
Source: PLoS One. 2022 Jul 27;17(7):e0271255. doi: 10.1371/journal.pone.0271255 (PMC9328529; doi:10.1371/journal.pone.0271255)
Supplement: S1 Table — (DOCX) [file pone.0271255.s002.docx]

**S1 Table. Reliability and validity of questionnaires.**

| **Reliability Statistics** | | | |
| --- | --- | --- | --- |
| **Questionnaire** | **Cronbach's Alpha (α)** | **Cronbach's Alpha Based on Standardized Items** | **Number of Items** |
| **Novaco anger Scale (NAS)** | .909 | .908 | 25 |
| **SSGS Guilt subscale** | .834 | .838 | 5 |
| **SSGS Shame subscale** | .756 | .766 | 5 |
| **Severity Measure for Depression (SMD)** | .868 | .868 | 9 |
| **Generalized Anxiety Disorder 7-item (GAD-7) scale** | .774 | .773 | 7 |
| **Personal Well-being Index – Adult (PWI)** | .841 | .843 | 7 |
